# Cost-Utility Analysis of Incobotulinumtoxin-A Compared With Conventional Therapy in the Management of Post-Stroke Spasticity in Romania

**DOI:** 10.3389/fphar.2019.01516

**Published:** 2020-01-16

**Authors:** Adina Turcu-Stiolica, Mihaela-Simona Subtirelu, Ana-Maria Bumbea

**Affiliations:** ^1^ Department of Pharmacoeconomics, Faculty of Pharmacy, University of Medicine and Pharmacy of Craiova, Craiova, Romania; ^2^ Department of Physical Medicine and Rehabilitation, Faculty of Medicine, University of Medicine and Pharmacy of Craiova, Craiova, Romania

**Keywords:** incobotulinumtoxin-A, post-stroke spasticity, conventional therapy, cost-utility analysis (CUA), upper-limb spasticity

## Abstract

**Objectives:** In Romania, the strokes’ incidence is of 61,500 per year and improving upper limb function is the essence in rehabilitation after a stroke to maximize the patient quality of life and reduce disability. In this study, it is compared the cost-effectiveness of the treatment of post-stroke upper limb spasticity with incobotulinumtoxin-A (INCO), with or without electromyographic control, against the conventional therapy programme alone (CON).

**Methods:** A Markov state transition model was developed to effectuate a cost-utility analysis (CUA). Measurements of health-related quality of life were derived from relevant clinical trials. Utility values for quality of life by response status were derived from the Short-Form-12 (SF-12) Health Survey data from a published study. The incremental cost-effectiveness ratio (ICER) of INCO (fixed, every 12 weeks) against CON was calculated in Ron per quality-adjusted life-year (QALY) gained for both therapies. Costs and outcomes were discounted using different scenarios at 3% and 5% per year with a time horizon of 3 and 5 years because Romanian legislative norms don’t specify the discount rates and time horizon for pharmacoeconomic analysis. Probabilistic sensitivity analyses (PSA) were managed on the base case with distributions attributed to the frequency of repeat dosing and utility valuation of the responder and the non-responder for health utilities derived from both mental and physical health state.

**Results:** Compared with CON, in all 4 scenarios, therapy with INCO had an incremental cost-effectiveness ratio (ICER) of less than 950 Euro per QALY gained (1 Euro = 4.7 Ron). INCO proved to be more favorable treatment option than CON in the treatment of upper limb spasticity in Romania. Despite costs being higher for patients treated with INCO, this treatment has more advantageous Incremental Cost-Effectiveness Ratio.

**Conclusions:** This therapy should be taken into account when considering rehabilitation options because it is highly cost-effective at < EURO 1,000/QALY gained, a very low WTP (Willingness To Pay) threshold. INCO proved to be a disruptive innovation because it is a new and more effective treatment, and, in the end, much higher in quality of life for patients with post-stroke upper limb spasticity.

## Introduction

Post-stroke upper limb spasticity is a challenging complication associated with stroke, with different incidence according to the different cerebral infarction site and to the period it appeared after stroke. The incidence of upper limb spasticity varies from 8.3% for the cerebral infarction site of the cerebellum to 63.3% for the cerebral infarction site of the basal ganglia and internal capsule (Jin and Zhao, 2018). The prelevance of spasticity during the first year after stroke varies from 17% at 12 months after stroke (Lundstrom et al., 2008) to 33% at 3 months after stroke (Kong et al., 2012).

Based on the findings of the King’s College London (Stevens et al., 2015), incidence estimate in Romania is of 190.9 strokes per 100,000 inhabitants annually age- and sex-adjusted, and prevalence estimate is of 833.3 strokes per 100,000 inhabitants age- and sex-adjusted, 252,774 strokes. The estimated number of new strokes in Romania in 2015 was 61,552 with an estimated increase in 2035 of 24%. Given these observations, urgent management of resources for post-stroke therapeutic strategies is needed.

Recovery of a stroke patient is difficult and goes through several stages, depending on its complications (Bumbea et al., 2013). Spasticity, as a complication following a stroke, is a movement disorder associated with increased muscle tone, improper limb posture, extreme contraction of antagonist’s muscles, and hyperactive cutaneous and tendon reflexes (Obrien et al., 1996). Health care costs are four times higher for stroke survivors with spasticity compared to those without spasticity (Harvey and Stein, 2014). Following a stroke, spasticity in the upper limb affects between 19% (Sommerfeld et al., 2004) and 65% (McGuire and Harvey, 1999).

Spasticity after stroke is usually met in the upper than the lower limbs and it was reported to be more frequent among younger than older patients (Sommerfeld et al., 2012). Post-stroke spasticity emerges in the first place at the elbow flexors and later in the elbow extensors, and continued with the wrist flexors (Opheim et al., 2014), limiting patients’ ability to eat, care for themselves, or perform other daily activities, being associating with a lower quality of life, greater treatment cost, and increased caregiver burden.

Various studies have assessed predictors that will help the identification of patients that are at risk for developing post-stroke spasticity. Lundstrom et al. (2010) found a higher risk for spasticity as early as 1 month after stroke is associated with high paresis of the arm (more than 2 points for item 5 of the NIHSS – National Institutes of Health Stroke Scale) at baseline. Smoking is associated with increased risk, and also a younger age of patients. Urban et al. (2010) stated that patients with a higher degree of paresis (BMRC - British Medical Research Council - grades 1 and 0) in the proximal and also in the distal muscles of the upper and lower limbs during the acute stage of the disease are more likely to develop post-stroke spasticity. The development of post-stroke spasticity in the upper and lower limbs was more likely to be encounted in subjects with initial hemihyperthesia than in others without sensory deficits like sensitivity to light touch. Severe spasticity was predicted by lower BI (Barthel Index) scores. Hemispasticity and raised muscle tone (Modified Ashworth Scale - MAS score ≥1) in more than two joints at every 6 weeks, combined with severe degrees of paresis at 16 weeks were considered risk factors for permanent state of spasticity (Wissel et al., 2010).

Common treatment interventions for spasticity change from conventional therapy with oral medications to a more aggressive one, like surgery. Treatments can vary depending on the severity of the spasticity. The common treatments for spasticity are the oral medication that blocks the neurotransmitters causing the muscles to tighten: baclofen (5–20 mg 3–4 times daily), benzodiazepine (such as diazepam, clonazepam), or tizanidine (4–36 mg daily). They are not a cure for spasticity and they relax all muscles even if they are affected by spasticity or not. Some commonly reported side effects of these oral medications are drowsiness, asthenia or weakness (Thibaut et al., 2013).

Rather than having an oral treatment that relaxes every muscle, having intramuscularly injected medications allows in a safe way, the affected muscle to be object of attention specifically with botulinum toxin (BoNT). Declining the spasticity in a patient’s muscles can permit them to join in physical therapy and finish rehabilitation programmes and exercises that will help them recover. Rehabilitation programmes could include: modified constraint-induced movement therapy (mCIMT) confronted with a neurodevelopment therapy programme; task practice therapy with cyclic functional electrical stimulation (FES) also confronted with only task practice therapy; and finally occupational or manual therapy with dynamic elbow extension splinting compared with only occupational therapy (Demetrios et al., 2013).

Botulinum toxin (BoNT) agents are administrated by intramuscular injection, without or with electromyographic control, for the treatment of localized spasticity treatment through inhibition of acetylcholine release at the neuromuscular junction, thereby reducing muscle contractions (Thibaut et al., 2013). Two antigenically distinct serotypes of BoNT are available on the pharmaceutical market as type A and B. Simpson et al. (2009) pointed out that BoNT-A is safer and more efficient than tizanidine, with a more reduction of muscle tone in upper limbs and lower incidence of side-effects. BoNT-A can have also a range of side effects, such as trouble swallowing, soreness, rash on the skin, and weak muscles.

Onabotulinumtoxin-A (Botox^®^) was first approved by FDA (the United States’ Food and Drug Administration) for the treatment of adults patients’ upper limb spasticity located at the elbow (biceps), wrist (flexor carpi ulnaris and radialis), and fingers (flexor digitorum profundus and flexor digitorum sublimis) (Thibaut et al., 2013). The marketplace now has another two botulinum neurotoxin type A products: one approved in 2009 - abobotulinumtoxin-A (Dysport^®^) and the other one approved in 2011 - incobotulinumtoxin-A (Xeomin^®^). Some studies have established comparative potencies between them. (Dashtipour et al. 2015) reviewed the literature and concluded that a dose conversion of 2.5U to no more than 3U of abobotulinumtoxin-A for each unit of onabotulinumtoxin-A provides an appropriate balance of safety and efficacy. The suggested doses per injection session to treat spasticity after stroke were up to 600 units (U) of onabotulinumtoxin-A and INCO or up to 1500 U of abobotulinumtoxin-A (Wissel et al., 2009).

The key contribution of BoNT-A in the management of post-stroke spasticity has been changed in the last few years, varying from muscle chemodenervation (nerve block) to grow into a more useful tool in order to improve limb posture, admitting hygiene, willing to stand, and walk, reducing spasticity-related pain (Santamato et al., 2016).

From all BoNT-S formulations, INCO is a150 kDA neurotoxin free from complexing proteins that could be an advantage, being relating to a lower risk of immunogenicity (Santamato et al., 2016).

In Romania, INCO (Xeomin^®^, Merz Pharmaceuticals GmbH) is licensed from 2014 for the symptomatic management of blepharospasm, cervical dystonia, and also post-stroke spasticity of upper-limb manifested by the joining of the hand in a position of flexion and clenched fist. The accurate dose and number of injection sites should be adjusted to each patient, depending on the size and location of the implicated muscles, the severity of spasticity, and the presence of local muscular weakness. In the pivotal clinical trial, the total and minimum doses were 170 units and respectively 400 units per treatment session. In general, repeated treatment should not be more frequent once every 12 weeks. Taking too much botulinum toxin doses may increase the risk of developing antibodies, which may cause treatment failure. Potential antibody formation may be reduced by the injection of the lowest effective dose at the highest time intervals according to the therapeutic indications.

Chancellor and Smith (2011) demonstrated that bacterial proteins play an important role in promoting an immune reaction with loss of effect and reduction of activity. In contrast with the other BoNT, INCO contains pure neurotoxin with free of complexing proteins, thereby INCO has a lower immunogenic potential.

The objective of this present study was to provide evidence of the cost-effectiveness of INCO compared with conventional antispastic therapy in patients with post-stroke spasticity, from the perspective of Romanian health care providers. The comparator was selected based on the standard of care for Romanian practice in the management of post-stroke spasticity. We aimed to prove INCO is a disruptive innovation because it is a new and more effective treatment than conventional therapy, and, in the end, much higher in quality of life for patients with post-stroke upper limb spasticity.

## Methods

Romanian decision-makers need a framework that will permit to make decisions regarding reimbursement drug lists taking into account a fixed budget and competing choices. To warrant the quality of economic evaluation, several European national agencies developed methodological guidelines for the design of pharmacoeconomic appraisals (van Lier et al., 2018). Romanian legislation does not specify any HTA tools as a cost-effectiveness analysis or cost-utility analysis for a pharmacoeconomic evaluation of a new drug. This is the reason, based on other countries’ expertise, we used different scenarios with different discounts for costs (3% or 5%) and different values for the time horizon (3 or 5 years).

### Model Structure

A Markov state transition model was elaborated in Microsoft Excel (Edlin et al., 2015) to perform a cost-utility analysis (CUA). Measurements of health-related quality of life were derived from relevant clinical trials. Utility values for quality of life by response status were derived from Short-Form-12 Health Survey (SF-12) data from a published study (Dressler et al., 2015a) that compared the efficacy and safety of INCO with CON for post-stroke arm spasticity. Mental Component Summary-12 and Physical Component Summary-12 are pointed as higher scores mean a better emotional and physical function, respectively, with the mean score being 50 and 10 for standard deviation in the general population. Responses from SF-12 were converted to utilities using a mapping algorithm (Sullivan and Ghushchyan, 2006). Quality-adjusted life-years (QALYs) were the measure of benefit, so health effects were expressed in terms of QALYs. These were calculated for two scenarios of time horizon: 3-year time horizon and 5-year time horizon. The Markov model followed patients in cycles of 12 weeks.

Dose limitation of 400 U for INCO was used (the mean = 363.1U, SD = 67.9U) (Kanovsky et al., 2011). The TOWER trial (Wissel et al., 2017) proved that an increase from 400 U up to 800 U enabled the treatment of a greater number of muscles and clinical spasticity models, following in reducing spasticity after stroke with increased improvements of muscle tone, without compromising patients’ safety or tolerability. Dressler et al. (Dressler et al., 2015b) demonstrated that INCO can be used safely in doses bigger than 400 U and up to 1,200 U (the mean 570.1 U ± 158.9 U) without detectable systemic toxicity. In the study that compared long-term efficacy and safety of INCO and CON of post-stroke spasticity, INCO doses were 215 ± 114 U at first intervention and 268.7 ± 155 U at the fifth intervention. Flexor digitorum profundus, flexor digitorum superficialis, and biceps brachii were the most often injected muscles.

We analyzed the outcomes with a lifetime horizon for estimating clinical and cost-effectiveness that reflect all important differences in costs or outcomes between INCO and comparator. Costs and outcomes were discounted using different scenarios at 3% and 5% per year with a time horizon of 3 and 5 years: scenario 3%/3years (a), scenario 5%/3years (b), scenario 3%/5years (c), scenario 5%/5years (d). We used sensitivity analysis to explore the impact of lower discount rates or longer time horizon.

Bayesian Markov chain Monte Carlo simulation methods are used to accomplish cost-effectiveness models. All variables were altered simultaneously for 5,000 iterations, with different distributions for cost inputs (gamma distributions), utility parameters (normal distributions), and rates of clinical events (beta distributions).

**Figure 1:**
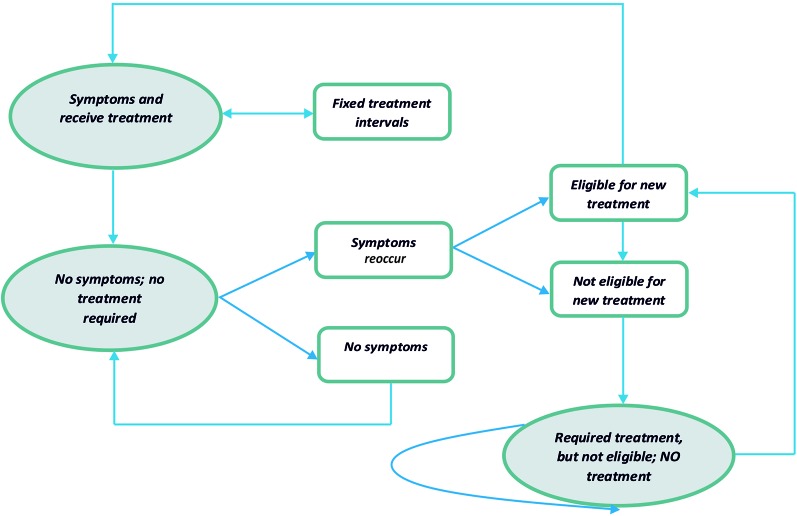
The structure of the Markov model.

The Markov model consists of three health states and transitions between them were reflected in **Figure 1**. Both types of treatment (with the INCO group and with conventional therapy group) were accepted to be effective and patients were moved to the state “no symptoms” or “required treatment, but not eligible.” One of the differences between the INCO and conventional therapy arms of the Markov model was the interval for every treatment (12 weeks and 4 weeks). If the patient was eligible for treatment based on the time, he or she would pass back to the original healthy state. There are no limits of numbers of cycles of treatments with INCO or conventional treatment in Romania.

Probabilistic sensitivity analysis (PSA) is most suitable for examining the joint parameter uncertainty in a model and to investigate overall robustness to changes in input values (Bringgs et al., 2012). PSA was managed on the base case with distributions attributed to the frequency of repeating dosing and utility valuation of the responder and the non-responder health for both mental and physical health state. The PSA was performed using 1000 iterations with Dirichlet distributions (for the proportion of the population demanding repeated injections and conventional treatments at each time interval) and beta distributions (for utility values).

### Costs

The model used direct costs to the health care system. It should be noted that lost productivity and leisure time costs were not accounted for. The direct costs included those of drugs (intramuscular injection vs oral medication); other antispastic medication; upper limb therapy sessions, provided by specialist physiotherapists; requiring hospitalization; and other health care and social services, including day-patient hospital treatment, nurse visits, therapy. The unit costs were from Romanian national sources. The costs were in Romania Ron, for the year 2017.

One vial of Xeomin contains 100 units of botulinum neurotoxin type A, purified from cultures of Clostridium botulinum (tulpina Hall) -150 kD, without proteins complex. The price of 1 vial of Xeomin is 570.4 Ron (1 Euro = 4.7 Ron). Transport of the botulinum neurotoxins in the muscle tissue is ensured by intramuscular injection of botulinum neurotoxin diluted with 0.9% NaCl/H_2_O (saline). An injection is performed by syringes with certain needle gauges (Kaymak et al., 2018). The injections are frequently multi-site, with 3 to 10 injected muscle groups. Muscles are localized by electromyographic stimulation, for each session it is allocated 30 to 45 min depending on the number of muscle treated and any technical complications. The injection sites’ number depends also on the structure and size of the muscles.

The paralysis therapy that generates spasticity is reimbursed in Romania (Contract-framework 2018–2019 of Romanian Ministry of Health, 2008). The therapeutical services cost for adults and children with a weight greater than 25 kg, on patient/3 months 1,461.05 Ron (with electromyographic control) or 1,245.12 Ron (without electromyographic control). The therapeutical services cost for children with a weight smaller than 25 kg, on patient/3 months 905.28 Ron (with electromyographic control) or 689.35 Ron (without electromyographic control). The costs of vials and all the services are included in these hospitalization costs.

For the first time, the upper limb spasticity therapy as a result of a stroke for the adult patient (with botulinum toxin) is reimbursed and the value is 1,999.36 Ron/3 months/patient. The data for antispastic medication used in actual practice were collected for the disease code 367, G24.8 or G24.9, from the Romanian Insurance House Database. Direct medical costs for conventional therapy were measured in Ron and based on a Romanian retrospective cost survey at National Health Insurance House.

Dressler et al., (2015a) compared the efficacy and safety of INCO with CON for post-stroke spasticity and concluded that muscle tone improved for all spasticity patterns between 63% and 86% (INCO) and 16%–27% (CON). Also, quality of life, measured by physical score and mental score, improved by 8.0 and 10.8 under INCO, and by 0.8 and 5.7, respectively, under CON.

## Results

### Benefits

The mapping algorithm transformed Mental Component Summary (MCS-12) and Physical Component Summary (PCS-12) scores from available values of SF-12 into preference-based HRQL scores as in **Table 1**.

**Table 1:** Results after mapping the EQ-5D index from the SF-12.

Utilities	Index	Incobotulinumtoxin-A		Conventional Therapy	
		Mean	SD	Mean	SD
Responder health state	Mental Score	52.9	11.0	41.4	12.5
	Physical Score	42.0	8.4	36.3	8.1
	EQ-5D	0.84	0.22	0.71	0.23
Non-responder health state	Mental Score	42.8	14.8	37.8	14.4
	Physical Score	33.6	7.8	35.5	9.3
	EQ-5D	0.7	0.25	0.68	0.26

### Costs

The model results for the four scenarios are shown in **Table 2**. Total costs in the INCO group are much higher than in the CON group because upper limb therapy sessions with INCO are provided by specialist physiotherapists and require hospitalization, other antispastic medication or other health care and social services, including day-patient hospital treatment, nurse visits.

**Table 2:** Cost-effectiveness of incobotulinumtoxin-A compared to conventional therapy and scenarios with different discount rates and time horizon.

Discount rate/ Horizon time	Costs (Euro)			QALYs			ICER (Euro/ QALY)
	INCO	CON	Incremental	INCO	CON	Incremental	
**Combined scenarios**							
3%/3years	3605.26	2524.56	1080.70	29.71	28.55	1.17	926
5%/3 years	5746.37	4085.21	1661.16	48.13	46.28	1.85	898
3%/5years	3508.89	2458.91	1049.97	28.89	27.76	1.13	927
5%/5 years	5492.80	3906.03	1586.77	45.95	44.18	1.77	899

### Cost-Effectiveness in Post-Stroke Spasticity of the Upper Limb

Compared with therapy alone, in all scenarios, therapy with INCO had an incremental cost-effectiveness ratio of almost 1,000 Euro per QALY gained. This therapy should be taken into account when considering rehabilitation options because it is highly cost-effective at < EURO1,000/QALY gained, a relatively low and conservative WTP (Willingness To Pay) threshold (Claxton et al., 2015).

### Probabilistic Sensitivity Analysis

Across the entire 1,000 iterations of the PSA, there was an incremental cost of Euro 1,052.35 (95% CI 1,047.44–1,057.25) in 3 years with 5% discount scenario. INCO was associated not only with more costs but also with more QALYs than conventional treatment: the incremental QALY gain was 1.14 (95% CI 1.10–1.17). No significant differences were obtained for all four scenarios (**Table 3**).

**Table 3:** Probabilistic sensitivity analyses (PSA) results.

Combined scenarios Discount rate/Horizon time	Incremental cost (Euro) Mean(95% CI)	Incremental QALYs Mean(95% CI)
3%/3 years	1083.14 (1078.09-1088.19)	1.17 (1.13-1.21)
5%/3 years	1052.35 (1047.44-1057.25)	1.14 (1.10-1.17)
3%/5 years	1665.12 (1657.18-1673.05)	1.86 (1.80-1.92)
5%/5 years	1590.54 (1582.971598.11)	1.77 (1.72-1.83)

**Figure 2:**
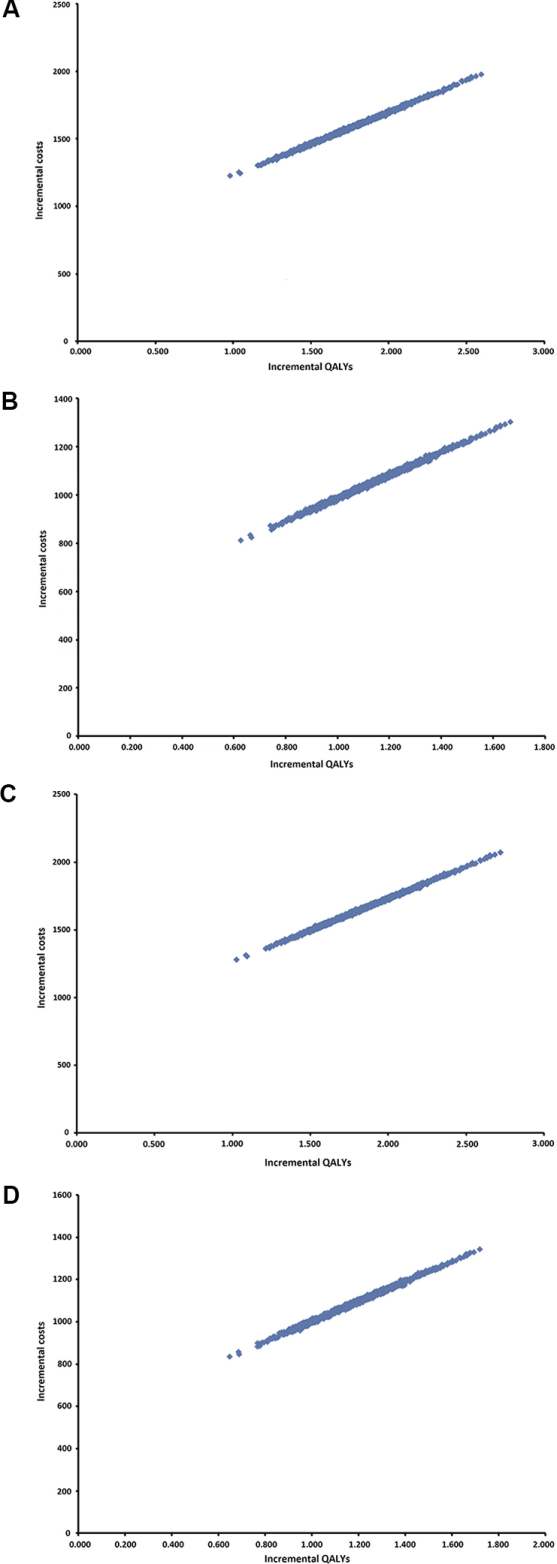
Probabilistic sensitivity analysis (PSA). **(A)** Scenario 3%/3 years. **(B)** Scenario 5%/3 years. **(C)** 3%/5 years. **(D)** 5%/5 years.

Results of the PSA are presented as an incremental cost-effectiveness scatterplot for all four scenarios (**Figure 2A–D**).

Quadrant 1 includes all 1,000 iterations and there are concluded that INCO is more effective and more costly than conventional therapy, for all four scenarios.

## Discussion

The current paper aimed to prove that INCO is a disruptive treatment that could replace conventional treatment because it improves stroke survivors function and quality of life for patients with post-stroke upper limb spasticity.

In summary, INCO proved to be more effective than CON in the treatment of upper limb spasticity in Romania, over 3 or 5 years as a lifelong time horizon. Despite costs being higher for patients treated with INCO, the INCO treatment shows a more favorable Incremental Cost-Effectiveness Ratio for both physical and mental health dimension. The QALYs gained over the 3-year time horizon or the 5-year time horizon were higher for fixed INCO, but the patients could alleviate their symptoms sooner and could not to wait till at least 12 weeks. The analysis and its results are reasonably robust as the PSA shows.

Kawalec et al. (2017) reviewed reimbursement environment in some Central and Eastern Europe countries, including Romania, and identified the implications of Health Technology Assessment (HTA) in the management of the public health care budget from Romania, where a scorecard HTA system is implemented. The drug policy in Romania is known to be centered on price reduction using various techniques and no value-based criteria are used. The pricing criteria is not included in the HTA (Health Technology Assessment) system, managed entry agreements are not used, and the main result is the absence of drugs for some therapeutic areas (Radu et al., 2018).

In Australia, INCO is licensed for flexible treatment intervals (at 6 weeks of minimum intervals and 20 weeks of maximum intervals) according to the patient’s clinical needs. The reimbursement of botulinum neurotoxin-A (BoNT-A) for treatment of moderate to severe post-stroke spasticity of the upper limb is limited to four treatment cycles per upper limb per lifetime. Makino et al. (2017) demonstrated continuing the INCO treatment over four cycles can be cost-effective, but the patient must be selected carefully from those with the major likelihood to continue to respond to many treatment cycles.

In Germany, a prospective, non-interventional, multicenter, parallel-group study was done to demonstrate the superior outcomes in muscle tone reduction compared to CON and significant improvement of the quality of life. Also, the cost-utility analysis favored INCO treatment (fixed, every 12 weeks) in comparison to CON alone and it was recommended with level A in national and international guidelines (Rychlik et al., 2016).

INCO may be considered clinically effective because it confers a net health benefit for patients with spasticity post-stroke, taking into account any adverse effects that could appear in the treatment with a botulinum toxin without complexing proteins. Dressler et al. (2012) reviewed the reported cases of antibody-induced therapy failure during the 5-year experience and demonstrated the hypothesis of an improved antigenicity. Even when applied in higher dosage, INCO did not produce antibody-induced therapy failure.

The insufficient attention paid to upper limb rehabilitation in the case of stroke patients must be resolved in Romania. Some other pharmacoeconomics studies were done to demonstrate a solution to this problem (Turcu-Stiolica and Subtirelu, 2018). Patients with upper limb spasticity could wash and dress alone, without leading to hygiene problems, infections, and pressure sores. The quality of their lives could increase and treatment of spasticity is one of the key components of stroke rehabilitation for improving voluntary movement and active function.

Societal benefits and costs were not taken into account and this is one of the limits of this study. Also, we did not have a QALY estimate for Romanian patients with post-stroke spasticity and there is a need for future researches about the quality of life of patients from Romania. But it is unlikely that QALY estimate for patients with post-stroke spasticity from two different countries to vary substantially enough to alter the conclusions of this analysis.

## Conclusions

A cost-utility analysis was lead to determine the incremental cost of the INCO per Quality-Adjusted Life Year (QALY) gained as compared to CON. We completed four scenarios, both for mental health and for physical health, that demonstrated INCO is cost-effective when compared with conventional therapy. Due to national different health care systems and varying approaches for the quality of life, there is a need for future studies about the assessment of costs and utilities in cost-effectiveness analysis in Romania. Our cost-utility analysis favored INCO treatment in comparison to CON. From our point of view, the results highlight the QALYs gained over differently selected time horizon for INCO. Using different scenarios at 3% and 5% discounts per year with a time horizon of 3 and 5 years did not lead to different outcomes.

## Data Availability Statement

The datasets generated for this study are available on request to the corresponding author.

## Author Contributions

AT-S coordinated and performed data analyses, developed the study protocol, reported study results, and drafted the manuscript. M-SS collected data and drafted the manuscript. A-MB coordinated data collection, analyzed study results, and revised the manuscript. All authors read and approved the final manuscript.

## Funding

AT-S received consultancy fees from Desitin Pharma to develop the cost-utility model, conduct the analysis and write the manuscript. The authors have no financial conflict with the subject matter discussed in the manuscript. Desitin Pharma had no role in the conception of, or decision to submit, this article.

## Conflict of Interest

The authors declare that the research was conducted in the absence of any commercial or financial relationships that could be construed as a potential conflict of interest.
